# Biodegradation of isoproturon by *Escherichia coli* expressing a *Pseudomonas putida* catechol 1,2-dioxygenase gene

**DOI:** 10.1186/s13568-023-01609-9

**Published:** 2023-09-26

**Authors:** Nagwa I. Elarabi, Abdelhadi A. Abdelhadi, Amr A. Nassrallah, Mahmoud S. M. Mohamed, Heba A. R. Abdelhaleem

**Affiliations:** 1 Department of Genetics, Faculty of Agriculture, Cairo University, Giza, 12613 Egypt; 2 Department of Biochemistry, Faculty of Agriculture, Cairo University, Giza, 12613 Egypt; 3 Botany and Microbiology Department, Faculty of Science, Cairo University, Giza, 12613 Egypt; 4 College of Biotechnology, Misr University for Science and Technology (MUST), 6(th) October City, Egypt; 5 Basic Applied Science institute, Egypt-Japan University of Science and Technology (E-JUST), P.O. Box 179, New Borg El- Arab City, 21934 Alexandria Egypt

**Keywords:** Biodegradation, Isoproturon, Catechol 1,2-dioxygenase, qPCR, *Pseudomonas putida*, *catA* gene

## Abstract

The phenylurea herbicides are persistent in soil and water, necessitating the creation of methods for removing them from the environment. This study aimed to examine the soil microbial diversity, searching for local bacterial isolates able to efficiently degrade the phenylurea herbicide isoproturon, 3-(4-isopropylphenyl)-1, 1-dimethylurea (IPU). The best isolates able to effectively degrade IPU were selected, characterized, and identified as *Pseudomonas putida* and *Acinetobacter johnsonii*. The catechol 1, 2-dioxygenase enzyme's *catA* gene was amplified, cloned, and expressed in *E. coli* M15. The Expressed *E. coli* showed high degradation efficiency (44.80%) as analyzed by HPLC after 15 days of inoculation in comparison to *P. putida* (21.60%). The expression of the *catA* gene in *P. putida* and expressed *E. coli* was measured using quantitative polymerase chain reaction (qPCR). The results displayed a significant increase in the mRNA levels of the *catA* gene by increasing the incubation time with IPU. Hydrophilic interaction chromatography (HILIC) mass spectrometry analysis revealed that three intermediate metabolites, 1-(4-isopropylphenyl)-3-methylurea (MDIPU), 4-Isopropylaniline (4-IA) and 1-(4-isopropylphenyl) urea (DDIPU) were generated by both *P. putida* and expressed *E. coli*. In addition, IPU-induced catA activity was detected in both *P. putida* and expressed *E. coli*. The supernatant of both *P. putida* and expressed *E. coli* had a significant influence on weed growth. The study clearly exhibited that *P. putida* and expressed *E. coli* were capable of metabolizing IPU influentially and thus could be utilized for bioremediation and biodegradation technology development.

**Supplementary Information:**

The online version contains supplementary material available at 10.1186/s13568-023-01609-9.

## Introduction

IPU is widely utilized as a phenylurea herbicide for post- or pre-evolution broad-leaved weed controls in cotton, wheat and fruit farms (Agbaogun et al. 2020). Hathout et al., (2022) data revealed that agricultural soil samples from Egypt governorates were highly contaminated (from 10 to 80%) by different pesticide residues. Many researchers have reported that IPU has deleterious effects on microbial communities and is carcinogenic for animals and humans (Vallotton et al. 2009; Alba et al. 2022). The IPU is documented to induce oxidative stress that results in the damage of proteins, lipids, and other cellular components, thus inhibiting growth or leading to organism death (Rutherford and Krieger-Liszkay 2001). Given these deleterious effects of IPU, it is necessary to reduce IPU pollution in water and soil. The detection of IPU as a source of water and soil pollution has encouraged studies to explore the natural microbial community able to metabolize IPU. Enrichment-culture methods have been utilized in the isolation of pollutant-degrading bacteria (ElGouzi et al. 2012; Mohamed et al. 2023).

Microbial biodegradation of herbicides in different environments is a cost-efficient, reliable and eco-friendly treatment method (Hussain et al. 2011; Wang et al. 2022). Pesticides, polyaromatic hydrocarbons, and heavy metals are some examples of pollutants that are currently being detoxified using microorganism-based products (Abdelhaleem et al. 2019; Elarabi et al. 2020, 2023; Chen et al. 2022). A set of IPU degrading microbes has recently been isolated from different polluted environments (Sun et al. 2009). Different types of bacterial species include both Gram-positive bacteria, for example, Bacillus and Rhodococcus (Arutchelvan et al. 2006; Elmelegy et al. 2023) and Gram-negative bacteria, such as *Achromobacter*, *Halomonas, Klebsiella* (Hinteregger et al. 1997), *Ralstonia* (Léonard et al. 1999), *Acinetobacter* (Hamzah and Al-Baharna 1994) and *Pseudomonas* (Agbaogun et al. 2020) have been recorded to degrade simple aromatic compounds, including benzoate and phenol. *Pseudomonas,* one of those bacteria, is known to be an effective biodegrading strain of herbicide phenolic compounds. In addition, *P. aeruginosa* strain JS-11 was reported as a promising isolate for IPU mineralization (Dwivedi et al. 2011).

Moreover, the genes and enzymes implicated in the biodegradation of the aromatic phenolic compounds have also been intensely clarified at the molecular level (Harwood and Parales 1996). The initial steps and processes involved have been documented in numerous studies, even if the whole IPU metabolic route is yet unknown (Sorensen et al. 2003; Sun et al. 2009). Demethylation of the dimethylurea side chain of IPU has been characterized as an initial, limiting step of IPU breakdown, leading to a temporary accumulation of MDIPU (3-(4-isopropylphenyl)- 1-methylurea) (Sun et al. 2009; Hussain et al. 2009). It has been hypothesized that the IPU metabolic pathway is started by two successive N-demethylations, then the urea side chain is cleaved, causing a brief accumulation of 4-isopropylaniline, and finally, the phenyl structure is mineralized (Sorensen et al. 2001; Hussain et al. 2009). Aerobically degraded pathways are generally utilized for aromatic phenol compound degradation through an intermediate or catechol, depending on the starting compound's chemical structure (Harwood and Parales 1996; Romero-Silva et al. 2013). Catechol 2, 3-dioxygenase splits the catechol adjacent to the hydroxyl groups through a meta-pathway, whereas catechol 1, 2-dioxygenase splits the catechol between two hydroxyl groups through an ortho-pathway (Zeyaullah et al. 2009). Catechol 1, 2-dioxygenase enzyme is considered the first enzyme of the β-ketoadipate pathway, a metabolic sequence utilized by microbes for the degradation of aromatic compounds (Aravind et al. 2021; Mohamed et al. 2023). Catechol 1, 2-dioxygenase, dioxygenase is a trivalent, nonheme, iron-consisting enzyme which stimulates the cleavage of the catechol aromatic ring to cis, cis-muconate with the integration of 2 atoms of oxygen into the substrate. In the process of mineralizing aniline by the IPU-degrading *Sphingobium* sp. strain YBL2, catechol and the first metabolite produced following phenyl ring cleavage, cis-muconic acid, were accumulated, according to Sun et al., (2009). Additionally, YBL2's *catA* gene, which catalyses the opening of the phenyl ring of catechol and codes for 1, 2-dioxygenase in various bacteria, was generated by PCR. It was suggested that catechol is a key intermediate in IPU mineralization as it is considered a key intermediate during the phenyl ring cleavage of aromatic compounds. Comparative studies of the regulation mechanisms in various microbial species indicate that there are different mechanisms of induction. Therefore, the organization of enzymes of the β-ketoadipate pathway is different in *Pseudomonas* (Patel et al. 1976) and *Acinetobacter* (Canovas et al. 1967). Catechol 1, 2-dioxygenases have been cloned and expressed in several bacteria (Nakai et al. 1995). The organization pathways of catechol 1, 2-dioxygenase (*catA*) genes have been recorded in *A. calcoaceticus* and *P. putida* (Parsek et al. 1992; Houghton et al. 1995). In addition, various isozymes of catechol 1, 2-dioxygenases were studied in *P. acidovorans* CA28, *Frateuria* sp. strain ANA-18, *P. arvilla* C-1 and *Pseudomonas* sp. strain B13 (Hinteregger et al. 1992; Nakai et al. 1995). This research was performed to obtain bacterial strains that could be appropriate for IPU biodegradation from IPU-treated agricultural soil. Furthermore, to isolate and express the catechol 1, 2-dioxygenase (*catA*) gene in *E. coli* M15.

## Materials and methods

### Chemical and soil sampling

IPU was purchased from Sigma-Aldrich^®^, Germany (cat. no. 34123-59-6). Analytical-grade IPU (chemical formula: C1_2_H_18_N_2_O and 99.5% purity). Two soil samples were taken from the superficial horizon (0–25 cm) from wheat soil treated with IPU situated at the experimental Faculty of Agriculture farm, Cairo University, Egypt (30.018121940665303, 31.208059082098913). The places of sample were chosen due to the contamination of soil with residual IPU.

### IPU degrading bacteria isolation

The IPU was prepared as a stock solution in acetone (10 mg/mL). Isolation and enumeration of the IPU degrading bacteria were performed utilizing the serial dilution method (1 g from the sample was disrobed in 100 mL sterilized dH_2_O and serially diluted to 10^–6^ with dH_2_O). Then, 1 ml of diluted suspension was added to 25 mL of mineral salts medium (MSM) medium supplemented with 50 mg/L IPU and incubated at 30 and 37 °C for 3 days. The bacteria were initially isolated using two different temperatures (30 and 37 °C) then the optimum temperature for the bacteria growth was chosen. The enrichment culture technique was utilized for IPU degrading bacteria isolation, where the IPU concentration was increased from 50 to 200 mg/L gradually in 100 mL MSM media. 1.0 mL bacterial culture was inoculated in MSM agar supplemented with 200 mg/L IPU. Bacterial colonies formed on the MSM plates after 72 h of incubation at 30 °C. Pure single colonies were selected.

### Minimum inhibitory concentration (MIC) determination

MIC was measured utilizing MSM supplemented with 50, 100, 150, and 200 mg/L IPU and incubated for 72 h at 30 °C.

### Morphological identification

Morphological characteristics were analyzed utilizing Bergey’s manual of systematic bacteriology (Sneath, 1986). The selected bacterial isolates according to colony forming unit (CFU) determination for IPU degradation were first characterized according to Gram staining, motility, colony morphology, and biochemical tests including catalase, methyl red, citrate utilization, indole production, urease dihydrolase, oxidase, and Voges Proskauer. The bacterial isolates were cultured at different temperatures (30 °C, 37 °C, 41 °C, and 44 °C) on Luria Broth (LB) media. For hemolysis detection, the Horse blood (5%) agar plates (biomérieux) were utilized.

### Molecular identification of the selected bacterial isolates

*16S rRNA* amplification by polymerase chain reaction (PCR) was utilized. Two universal primers (27F and 1492R) were utilized for *16S rRNA* fragment 1520 bp amplification (Additional file 1: Table S1) (Abdelhadi et al. 2016). One percentage agarose gel was used for PCR reaction visualization under UV light. The phylogenetic trees were conducted using Clustal Omega from The EMBL-EBI analysis tools (https://www.ebi.ac.uk/Tools/msa/clustalo/).

### Primer design of *catA* gene amplification

Based on the DNA sequence of *P. putida catA* gene in the national center for biotechnology information (NCBI) Database (accessions: D37782.1, D37783.1, EU000397.1 and MN442426.1 on www.ncbi.nlm.nih.gov), the primers of *catA* gene isolation responsible for IPU degradation were designed utilizing the OligoPerfect™ Designer tool (https://tools.thermofisher.com/content.cfm?pageid=9716). The amino acid analysis was done using the Expasy ProtParam tool (https://web.expasy.org/protparam/).

### PCR

The reaction was executed in 50 µL total volume including 50 ng/μL of isolated DNA (5 μl) from each sample, 2 μM/μL of both primers (2.5 μl), 25 μl GeneDireX^®^ One PCRTM (Cat. no.MB203-0050) master mix and 15 μl sterilized dH_2_O. The bacterial DNA template was prepared using Simply™ Genomic DNA extraction kit (GeneDirex, Inc, Taiwan, cat. no. SN023-0100) according to the manufacturer’s instruction manual. For PCR conditions, denaturation at 94 °C for 5 min; 40 cycles of denaturation at 94 °C for 1 min; 1 min of annealing following Additional file 1: Table S1; 2 min of extension at 72 °C; and 7 min of extension at 72 °C. Wizard^®^SV Gel and PCR Clean-up System (Promega, USA, cat. no. A9280) was used for the PCR cleanup, and sequencing was carried out at Macrogen Company in Korea.

### Expression of *catA* gene in *E. coli* M15

*E. coli* M15 strain QIAexpress was purchased from Qiagen, USA. DNA was extracted from *P. putida* FACU (accession number LC425130.1) using genomic DNA isolation kit (GeneDirex, Inc, Taiwan, cat. no. SN023-0100). The *catA* open reading frame (OPF) was amplified by PCR utilizing the catA-*Bam*HI and catA-*Hind*III primers (Additional file 1: Table S1). A *Bam*HIsite was introduced to the forward primer whereas a *Hind*IIIsite was introduced to the reverse one. The amplified product was digested with *Bam*HI (FastDigest *Bam*HI, Thermo scientific^™^, cat. no. FD0054) and *Hind*III (FastDigest *Hind*III, Thermo scientific™, cat. no. FD0504)*.* Then, the 936 bp DNA fragment was cloned into a pQ30 vector (N-Terminus pQE Vector Set, Qiagen, USA, cat. no. 32915) that digested with the same restriction enzymes followed by T4 DNA Ligase (Thermo Fisher Scientific, USA, cat. no. EL0011) reaction. The resulting constructs, designated pQ30-*catA*using snapgene software (Additional file 1: Fig. S1). Heat shock transformation was used to introduce the recombinant vector into the *E. coli* M15 strain.

### Sodium dodecyl sulfate–polyacrylamide gel electrophoresis (SDS-PAGE)

Cell extracts were mixed with SDS-extraction buffer (10% [v/v] 2- mercaptoethanol, 4% [w/v] SDS and 150 mM Tris–HCl pH 6.8). The extractions were boiled for five min and centrifuged at 10,000 g at 4 ºC for 15 min. 0.25% coomassie brilliant blue R-250 (Bio-Rad) was utilized for gel staining and 10% acetic acid and 7% methanol were utilized for gel destaining. 15% (w/v) SDS-PAGE was utilized for proteins (50 mg) analysis (LaemmLi 1970).

### Inoculum preparation for enzyme activity

*P. putida, E. coli* M15 and expressed *E.coli* strains were grown in 100 mL of LB media and incubated at 30 and 37 °C for *P. putida* and *E. coli,* respectively. Bacterial cultures were centrifuged at 6000 rpm for five min and washed with MSM two times. The bacterial pellets were suspended in MSM and the optical density (OD) was adjusted to 1.0at 600 nm. About 1 mL of bacterial culture was inoculated into 150 mL MSM with 200 mg/LIPU as a sole source of carbon. The incubation temperatures were 30 and 37 °C for *P. putida* and *E. coli,* respectively with shaking 150 rpm for 15 days (Giri et al. 2016; Abdelhaleem et al. 2019).

### Biodegradation efficiency

Biodegradation efficiency of *P. putida,* expressed *E. coli* and *E.coli* M15 strains was determined by calculating the remaining IPU in MSM media after inoculation at intervals times; to residual IPU extraction, 50 mL aliquots were taken at 1, 5, and 10 days incubation from MSM and centrifuged to remove any cell from the supernatant. To evaluate the biodegradation efficiency, residual IPU was isolated by adding 50 mL of dichloromethane to an equal volume of cell free supernatant. The isolation steps were repeated three times. Anhydrous Na_2_SO_4_ was utilized to dry the extract and then the solvent evaporated to dryness. The residues were dissolved in water and 1.0 mL of acetonitrile (25/75, v/v). Acrodisc syringe filter (0.2 µ membrane) was utilized for sample filtration. The IPU extracts were injected into HPLC utilizing model Waters 2690 Alliance HPLC system supported by a Waters 996 photodiode array at wavelength 243 nm. The separation condition was column C18 5 µm X 250 X 4.6 mmand injected volume 2.0 µL. Samples were eluted from the column utilizing water and acetonitrile (25:75 v/v) at a flow rate of 1 mL/min. The HPLC analysis was carried out at room temperature. Residual IPU concentration was quantified utilizing a standard curve plotted between the IPU knew concentration and mass absorbance unit (mAU) (Giri et al. 2017). All the HPLC solvents (the dichloromethane cat. number 75-09-2 MERK Germany and the acetonitrile cat. number 75-05-8 MERK Germany) were HPLC grade from MERK, Germany (Manohar et al. 1999).

### Enzyme purification

MSM media including 200 mg/L IPU were utilized for enzyme extraction. 50 mL MSM media was inoculated with 1 mL of bacterial cultures and incubated at 30 and 37 °C for *P. putida* and *E. coli*, respectively for 5 days. After five days, the biomass was centrifuged, and the supernatant underwent protein precipitation. Saturation of ammonium sulfate (50 to 70%) was utilized for enzyme precipitation. In five mL of 0.05 M phosphate buffer (pH 7.5), the precipitant enzyme was recovered and re-suspended. Gel filtration chromatography (DEAE Sephadex A-50, 80 × 2 cm) was utilized for enzyme purification. The same buffer was utilized for column elution with gradient sodium chloride ranging from zero to 1 M at a flow rate of 0.1 mL/min. Fractions were eluted from 480 to 500 min. Dioxygenase activity and total proteins were measured in each collected fraction. Fractions that had catechol 1,2 dioxygenase activity were gathered, precipitated with a second batch of 50% to 70% ammonium sulphate, and then washed with distilled water using a dialysis tube. SDS-PAGE was used to analyze enzyme purity and collected for further tests. Protein concentration was measured utilizing bovine serum albumin as a standard (Lowry et al. 1951). All purification steps were performed at 4 °C.

### CatA kinetic constants determination

Michaelis–Menten constant (Km) and Maximum velocity (V max), the catalytic parameters, for the enzyme isolated from expressed *E. coli* were studied by determining the enzymatic reaction initial linear rates. After adding catechol in a range of concentrations (0–120 mM) at 35 °C, the catechol 1, 2-dioxygenase activity was determined (260 nm) by quantifying the production of cis, cis-muconic acid (CCMA), which has a molecular weight of 16800 M^−1^ cm^−1^. 40 mL of catechol (50 mM), 134 mL of Na_2_EDTA (20 mM), 1786 mL of phosphate buffer pH 7.5 (50 mM), and 40 mL of culture supernatant made up the reaction mixture used (Hegeman 1966). There were three measurements made for every substrate concentration. Based on the Michaelis–Menten equation, Km and V max were calculated.

### Solid-phase extraction (SPE)

After the end of incubation, the media were centrifuged and filtrated using Millipore filter disc 0.2 mm. Then the media was subjected to solid phase extraction SPE using super clean SPE columns (Phenomenex, Strata-X 33 um Polymeric Sorbent. part number 8B-S100-FBJ, 200 mg/3 mL). Three mL of methanol was utilized for column activation and then equilibrated with distilled de-ionized water. The media (250 mL) was purified and used in the SPE column. The elution rate was 10 drops/min, the IPU and its degradation derivatives were collected into the column matrix. In the end, the IPU was eluted using 1 mL of methanol and collected in a dark vial for further analysis.

### LC mass spectrometry

Liquid chromatography-electrospray ionization-tandem mass spectrometry (LC–ESI–MS/MS) with a SCIEX Triple Quad 5500 + MS/MS system outfitted with electrospray ionization (ESI) for detection and an Exion LC AC system for separation were used to characterize degraded derivatives of IPU inoculated or not with the aforementioned strains. The separation proceeded with an Ascentis® C18 Column (4.6 × 150 mm, 3 µm). The mobile phases contained two eluents A: acetonitrile; B: 0.1% formic acid (LC grade). The gradient for the mobile phase was programmed as follows: 10% B at 0–1 min, 10–90% minutes 1 through 33, 90% minutes 34 through 37, 10% minutes 37.1 through 40. The injection volume was 10 L, and the flow rate was 0.7 mL/min. Positive ionization mode was used for the MS/MS analysis with an EMS-IDA-EPI scan from 100 to 1000 Da for MS1 with the following parameters: Ion source gas 1 and 2 were 45 psi and from 50 to 800 Da for MS2, with a de-clustering potential of 80, collision energy of 35, and collision energy spread of 20. Curtain gas was 25 pressures, IonSpray voltage was 5500, and the source temperature was 500 °C. Using MS-DIAL software version 4.70 and Fiehn HILIC as an identification library for Alignment, compounds were identified (Meirinho et al. 2021).

### RNA extraction and qPCR

*P. putida* and expressed *E. coli* were grown on nutrient broth media for 24 h. The RNA from both *P. putida* and expressed *E. coli* was extracted utilizing the Enzymatic Lysis of Bacteria protocol of the QIAamp RNeasy Mini kit (Qiagen, Germany, cat. no. 74104). qPCR was used to measure messenger RNA expression levels of *catA* gene. Primer sequences are shown in Additional file 1: Table S1. The 25 µL reaction was consisted of 0.25 µL of RevertAid Reverse Transcriptase (200 U/µL) (Thermo Fisher), 12.5 µL of the 2 × QuantiTect SYBR Green PCR Master Mix (Qiagen, Germany, cat. no. 204343), 0.5 µL of each primer of (20 pmol), 3 µL of RNA template and 8.25 µL of water. The reaction was carried out in the Stratagene MX3005P qPCR machine. The stratagene MX3005P software was utilized for the Ct values and curves determination. The qPCR reaction was done under the following conditions: denaturation for ten min, at 95 °C, then 40 cycles of denaturation 15 s at 95 °C, annealing 30 s at 56 °C and extension 30 s at 72 °C.

### The effect of CatA on weed germination

To evaluate the ability of *P. putida* FACU *catA* gene for IPU degradation, MS media (Murashige and Skoog 1962) supplemented with different bacterial supernatants (MS free as a control, *P. putida* FACU supernatants, expressed *E. coli* supernatants, *E. coli* M15 supernatants and MSM medium without bacteria supernatants) were used after filtering by MS^®^ sterile syringe filter. *Capsella bursa-pastoris* and *Phalaris canariensis*, two different kinds of weed seeds, were sterilized using ethanol 70% for one minute and 50% Clorox (hypochlorite sodium) for thirty minutes before being washed three times with sterilized distilled water. On MS media with several kinds of bacterial supernatants, ten seeds were plated. After one week, the germination percentage was recorded. All experiments were repeated three times.

### Statistical tests

Utilizing the Graph Pad Prism 7 for Windows 10 computer software program and the Dunnett’s multiple comparisons test at a significance level of 0.05, a one-way analysis of variance (ANOVA) was carried out. To evaluate the RNA level of the bacterial strains, the Ct of each bacterial treated sample (with IPU) was compared with the untreated sample (without IPU) utilizing the following ratio: (2^−∆∆ct^) (Livak and Schmittgen 2001; Yuan et al. 2006).

While

$$\Delta {\text{Ct }} = \, \Delta {\text{Ct}}_{{\text{treated sample}}} {-} \, \Delta {\text{Ct}}_{{\text{untreated sample}}}$$

$$\Delta {\text{Ct}}_{{{\text{treated}}}} \, = \,{\text{Ct}}_{{catA\,{\text{gene}}}} - {\text{Ct}}_{{{\text{reference}}\,{\text{gene}}}} \,{\text{and}}\,\Delta {\text{Ct}}_{{{\text{untreated}}\,{\text{sample}}}} \, = \,{\text{Ct}}_{{catA\,{\text{gene}}}} - {\text{Ct}}_{{{\text{reference}}\,{\text{gene}}}}$$

## Results

### Isolation and identification of IPU degrading bacteria

The minimal inhibitory concentration for bacterial growth was determined to be 200 mg/L IPU based on the growth of the bacteria on MSM media with various concentrations of IPU. Ten IPU degraded bacterial isolates (IPU-FACU1 to IPU-FACU10) have been selected and purified. By adding 200 mg/L of the active ingredient of IPU to the incubation MSM medium, the susceptibility of bacterial isolates to degraded IPU was investigated. IPU-FACU1 and IPU-FACU7 bacterial isolates showed the highest ability to degrade IPU according to the CFU (Additional file 1: Fig. S2).

The IPU-FACU1 and IPU-FACU7 bacterial isolates were initially identified using morphological observation and physiological characteristics method. IPU-FACU1 and IPU-FACU7 were Gram-negative, catalase positive, rod-shaped, non-spore-forming, and formed cream and round colonies on LB agar. They did not reduce starch. IPU-FACU1 and IPU-FACU7 were grown at 30 °C, 37 °C and 40 °C. IPU-FACU1wasmotile but IPU-FACU7 was not-motile (Additional file 1: Table S2). The IPU-FACU1 isolate was classified as *Pseudomonas* sp. based on morphological and physiological identification, while the IPU-FACU7 strain was identified as *Acinetobacter* sp.

**Fig. 1:**
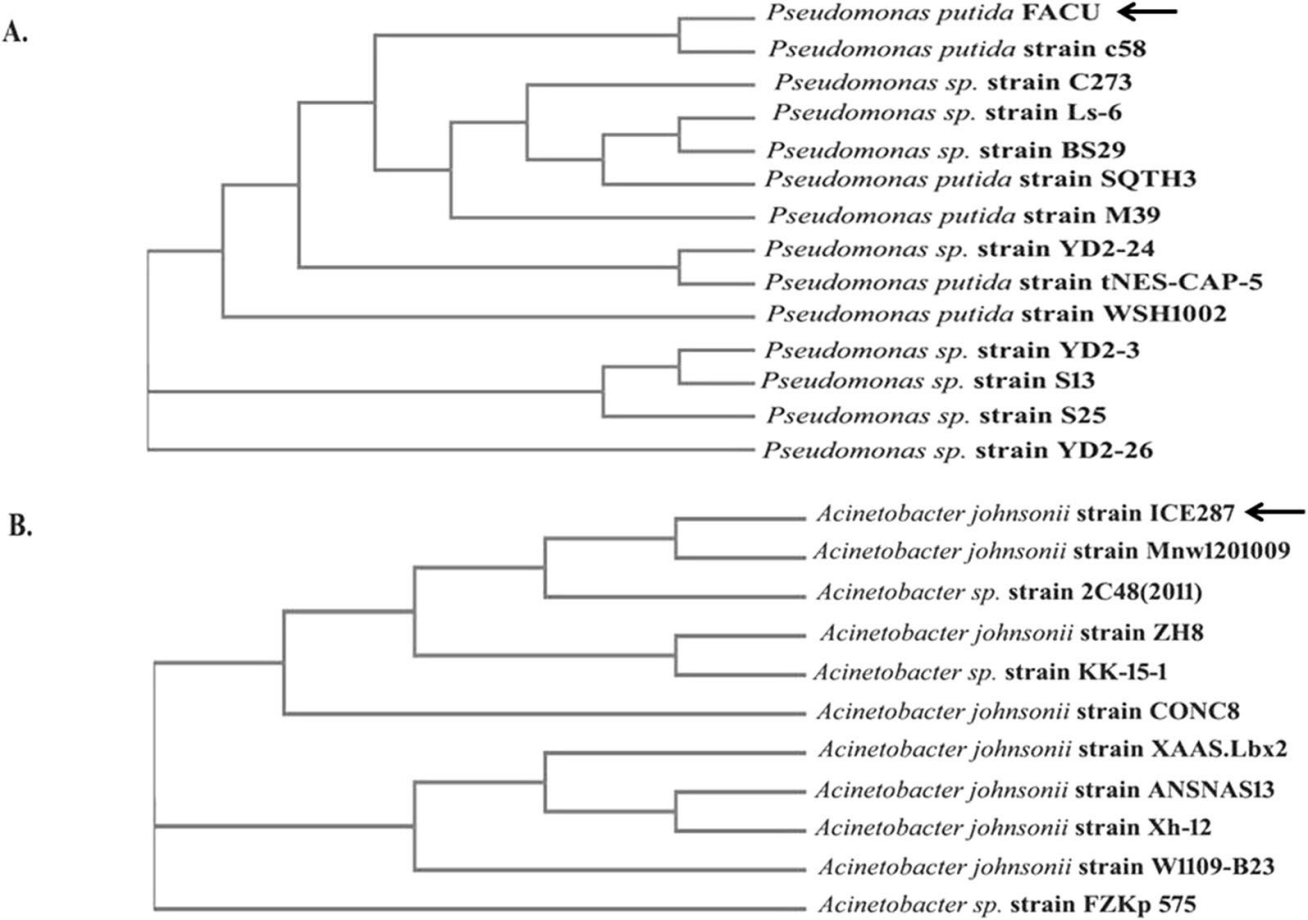
The phylogenetic tree for the *16S RNA* gene from IPU-FACU1 and IPU-FACU7 isolates. **A**: the phylogenetic tree of *P. putida* and **B**: the phylogenetic tree of *A. johnsonii.* The phylogenetic trees were constracted using Clustal Omega from the EMBL-EBI analysis tools (https://www.ebi.ac.uk/Tools/msa/clustalo/)

The *16S rRNA* gene was utilized for the molecular identification of the two bacterial isolates. About 1500 bp fragment was amplified, sequenced and aligned with other *16S rRNA* sequences on NCBI databases for both IPU-FACU1 and IPU-FACU7 isolates (Additional file 1: Fig. S3). The isolated IPU-FACU1, IPU-FACU7 displayed a high degree of sequence similarity with *16S rRNA* genes from *Pseudomonas putida* and *Acinetobacter johnsonii* (88.17 and 98%), respectively. The obtained sequence was submitted to DNA Data Bank of Japan (DDBJ) under accession numbers LC425130.1 and LC324680, as *Pseudomonas putida* (IPU-FACU1 isolate) and *Acinetobacter johnsonii* (IPU-FACU7 isolate), respectively. The BLAST results for *16S rRNA* nucleotide sequence were utilized to construct the phylogenetic tree for both bacteria strains (Fig. 1).

### Isolation of *catA* gene

The *catA* gene encoded for catechol 1, 2-dioxygenase enzyme of *P. putida* was involved in IPU degrading*.* NCBI available data for *P. putida* multi coding sequence of *catA* genes were utilized for primers design. The different sequences were aligned for selected conserved sequences of the *catA* gene. Two highly conserved sequences were detected, *catA* forward (F) and *catA* reverse (R) primers, to amplify *catA* ORF (Additional file 1: Table S1). In this research, PCR was used to amplify the coding sequence of *catA* gene (936 bp). Only *P. putida* showed an expected band (Additional file 1: Fig. S4).

**Fig. 2:**
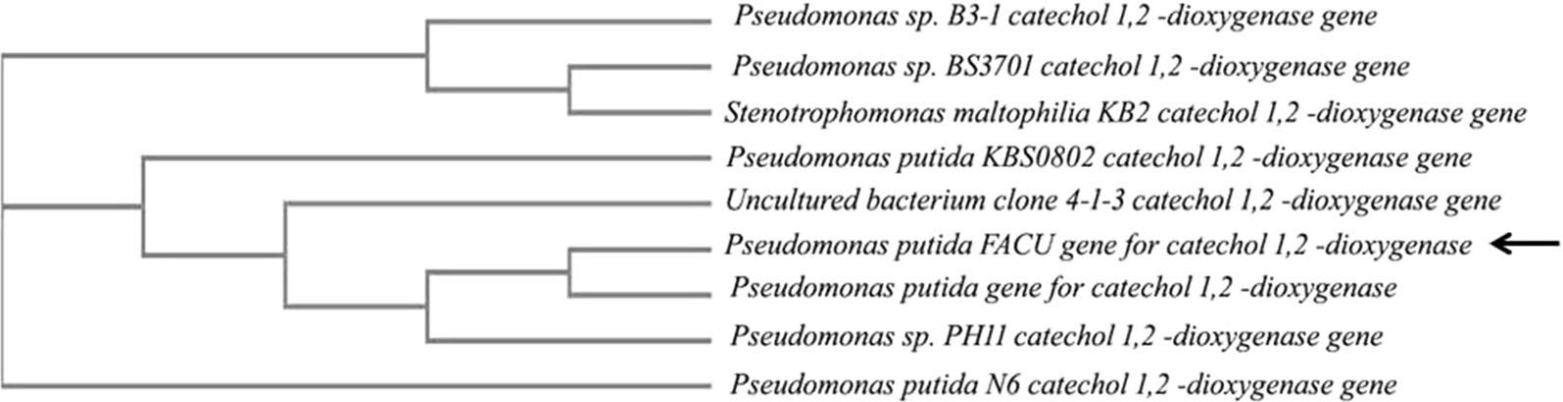
The phylogenetic tree of *P. putids* IPU-FACU *catA* gene based on nucleotide sequence BLAST. The phylogenetic trees were constructed using Clustal Omega from The EMBL-EBI analysis tools (https://www.ebi.ac.uk/Tools/msa/clustalo/)

PCR product for *catA* gene was sequenced and analyzed by blasting with available sequences in the NCBI. *P. putida catA* gene showed a high level of sequence similarity (100%) with *catA P. putida* gene for catechol 1, 2-dioxygenase accession number D37782.1. *catA* gene sequence was published in the NCBI with accession numbers LC426020. The BLAST result for the nucleotide sequence of *catA* gene was applied to construct the phylogenetic tree (Fig. 2). The bacterial strains were deposited and available in Culture Collection Ain Shams University (CCASU WDCM1186, Cairo-Egypt), under the numbers CCASU-2023-57 and CCASU-2023-58 for *A. johnsonii* and *P. putida* FACU.

From catA amino acid analysis, the *P. putida* catechol 1, 2-dioxygenase protein was composed of 311 amino acids with 34.26 KDa molecular weight and 5.21 isoelectric point. The negatively charged residues (Aspartic + Glutamic) were 46 while the positively charged residues (Arginine + Lysine) were 32. The high amino acid composition was alanine (11.6%).

### Expression of *P. putida* FACU *catA* gene into *E. coli* M15

*P. putida catA* ORF was amplified using PCR with catA-*Bam*H and catA-*Hind*III primers. The amplified fragment was cloned into pQ30 expression vector using *Hind*III–*Bam*HI site resulting in the pQ30-*catA* vector. The recombinant vector was transformed into *E. coli* M15 strain to identify the expression protein of the *P. putida catA* gene. Moreover, plasmid miniprep and the treatment of restriction enzymes were used for *E. coli* transformation checking. The expression optimum conditions for *catA* gene in *E. coli* were tested. Several isopropyl β-D-1-thiogalactopyranoside (IPTG) concentrations (0.1, 0.5 and 1 M) were used for protein induction (Additional file 1: Fig. S5A). The 1 M IPTG showed a high amount of protein. By SDS-PAGE analysis, pQ30-*catA* transformed in *E. coli* cells displayed increased levels of catA polypeptide under different incubation times (0.5, 1, 1.5, 2, 3, 4 h at 100 mM IPTG), compared to the wild type P. putida cells (Additional file 1: Fig. S5). In both cases, a minimum time of three hours after induction produced a substantial amount of protein (Additional file 1: Fig. S5B) longer induction did not result in any significant increase in yield. The protein is also studied at different temperatures (20, 28, 30, 37, 40 and 45 °C) for 3 h at 100 mM IPTG (Additional file 1: Fig. S5C). The results indicated that the best conduction for catA induction was 100 mM IPTG after 3 h of incubation at 37 °C.

### The IPU degradation efficiency

**Fig. 3:**
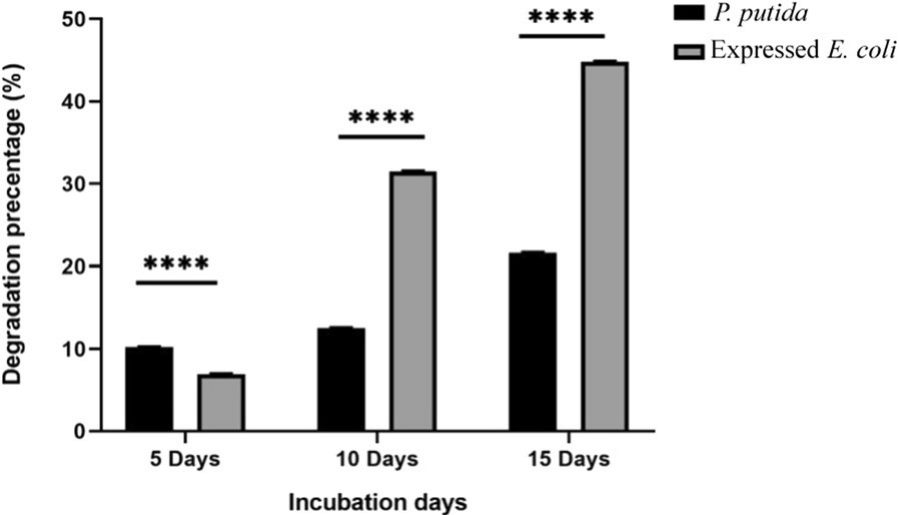
The biodegradation efficiency of IPU by *P. putida* and expressed *E. coli* (recombinant 1, 2 catechol dioxygenase) after 5, 10 and 15 days of incubation

The IPU degradation efficiency was determined using HPLC analysis*.* As result, *P. putida* FACU strain showed high degradation efficiency in the first five days of inoculation (10.19%) in comparison to *E. coli* expressed *catA* gene (6.9%). However, after 10 and 15 days of inoculation, *E. coli* expressed *catA* gene showed high degradation efficiency reach (31.48 and 44.80%) respectively in comparison to *P. putida* which reach (12.50 and 21.60%) in 10 and 15 days of inoculation (Fig. 3).

### Purification of the 1, 2 catechol dioxygenase

**Fig. 4:**
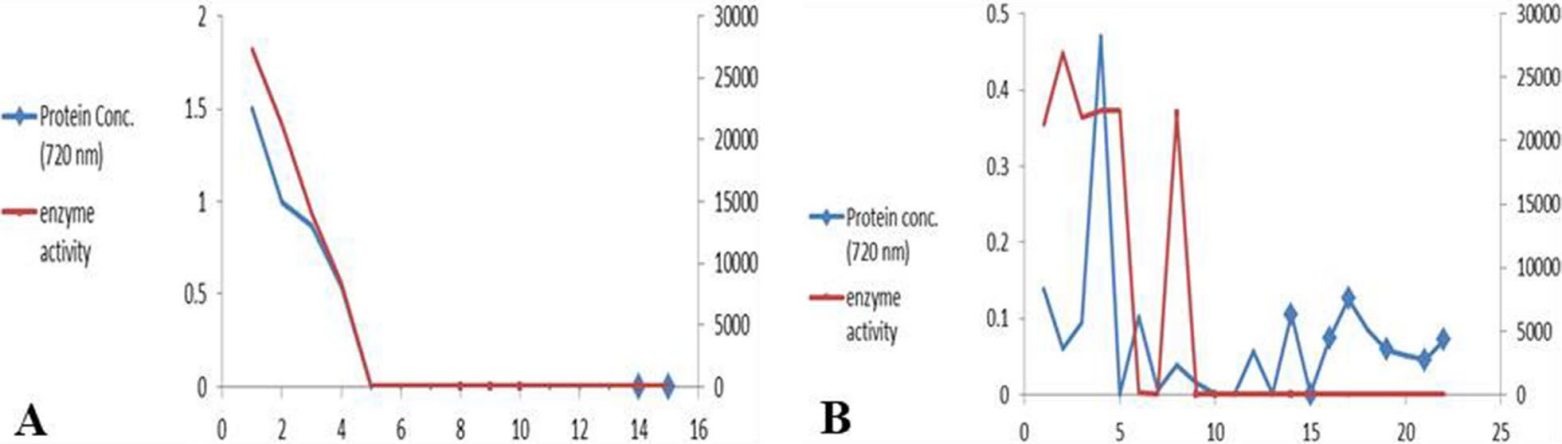
Purification of 1, 2 catechol dioxygenase using anion exchange/size exclusion chromatography on a Sephadex A-50 column from (**A**)*P. putida* and (**B**) expressed *E. coli.* Proteins from a crude bacterial culture that were 50–70% (NH_4_)_2_SO_4_ saturated were added to a column (802 cm) that had been equilibrated with 0.05 M phosphate buffer (pH 7.0). With a gradient of 0–1 M of NaCl and a flow rate of 1 mL/min, elution was performed

In Fig. 4 and Table 1, the size-exclusion enzyme purification chromatogram from expressed *E. coli* and *P. putida* was displayed. Four fractions from a total of fifteen fractions and five fractions from total twenty- two fractions from *P. putida* and expressed *E. coli* respectively had displayed enzyme activity. 86.7 and 80.2% of the 1, 2 catechol dioxygenase activity from the crude preparation was collected and yielded about 61.5 and 66.8%-fold increase in particular activity of enzyme isolated from expressed *E. coli* and *P. putida* respectively.

**Table 1:** Purification of 1, 2 catechol dioxygenase from expressed *E.coli* and* P. putida*

Step	Volume (mL)	Total protein (mg)		Total activity (U)		Specific activity (U/mg)		Yeild		Purification Factor	
		*E. coli*	*P. putida*	*E. coli*	*P. putida*	*E. coli*	*P. putida*	*E. coli (%)*	*P. putida (%)*	*E. coli*	*P. putida*
Crude preparation	50	60.3	32.4	631.4	243.6	10.4	7.5	100	100	–	–
(NH_4_)_2_SO_4_	5	13.3	9.5	547.5	195.4	41.16	20.5	86.7	80.2	3.9	2.7
DEAE Sephadex	5	4.5	4.3	336.9	130.7	74.8	30.3	61.5	66.8	1.8	1.4

### SDS PAGE for 1, 2 catechol dioxygenase enzyme

**Fig. 5:**
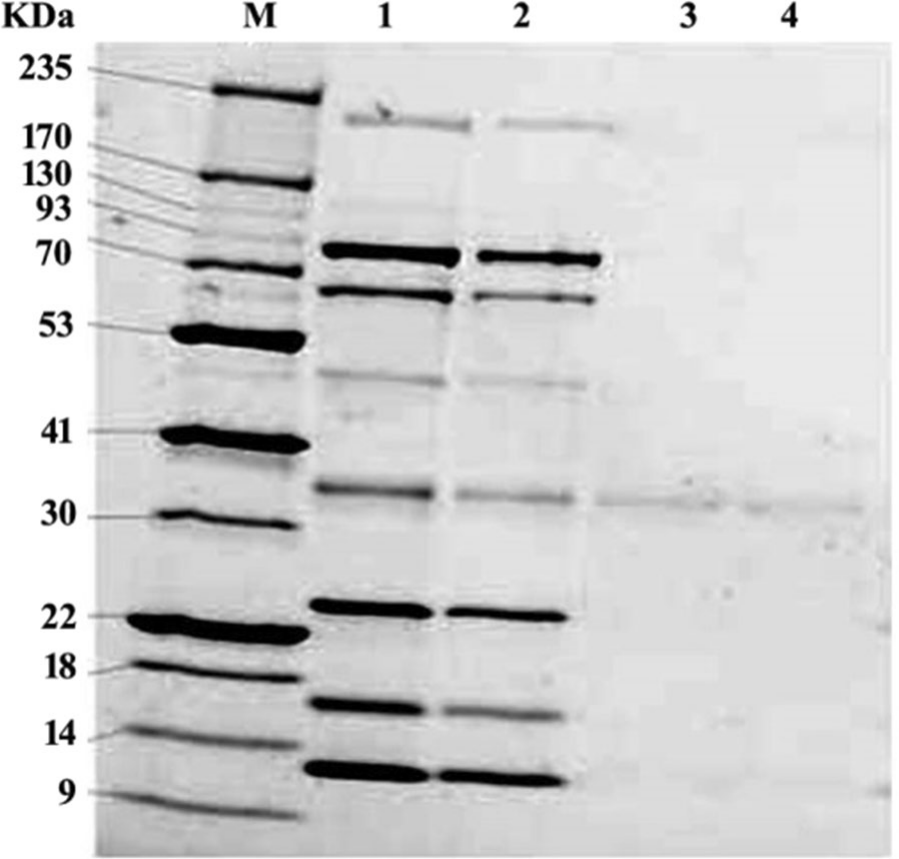
The 12% SDS-PAGE profile under denaturation conditions protein fractions obtained from anion exchange/size-exclusion chromatography of crude and recombinant catechol 1,2 dioxygenase from *P. putida* and expressed *E. coli* respectively. Lane M, Blue Starprestained protein marker, lanes 1 and 2 were crude protein from *P. putida* and expressed *E. coli*, lane 3 and 4 the purified enzyme from *P. putida* and expressed *E. coli*

Fractions with 1, 2 catechol dioxygenase activity were collected from *P. putida* and expressed *E. coli* and subjected to SDS-PAGE (Fig. 5). Each fraction displayed only one band with the same molecular weight (34.26 KDa); indicating that, the native and recombinant 1, 2 catechol dioxygenase, are similar fractions.

### Determination of K_m_ and V_max_

Enzyme kinetic determination was targeted to predict how enzymes act in living organisms. The kinetic constants were defined as K_m_ and V_max_. K_m_ was used in determining enzyme–substrate interaction; the strength of the enzyme–substrate complex (ES) and V_max_ reflected the velocity of the enzyme to catalyze the reaction so enzyme with a high K_m_ has a low affinity for its substrate and requires a greater concentration of the substrate to achieve V_max_.

**Fig. 6:**
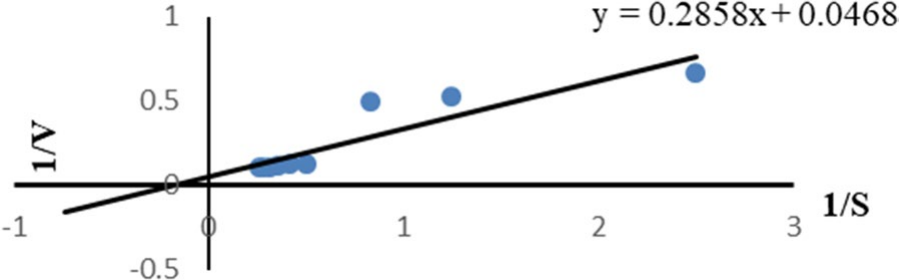
Determination of Km of the catA from *E. coli*. Km was measured by Lineweaver–Burk plot, (S is expressed in U/mg), utilizing catechol as a start material and determined as OD/mg protein/h at 720 nm

Enzyme kinetics was determined according to Line weaver–Burk plot where the inverse of the reaction rate (1/V) was plotted against the inverse of substrate concentration (1/S) as it showed in Fig. 6. The y-intercept was equivalent to the inverse of V_max_ and the x-intercept of the graph represents − 1/K_m_. The best activity of catechol 1,2 dioxygenase (V_max_) isolated from expressed *E. coli* was 21.36 U/mL (4.7U/mg) obtained at concentration (K_m_) 6.1 µM (Fig. 6).

### LC mass spectroscopy

**Fig. 7:**
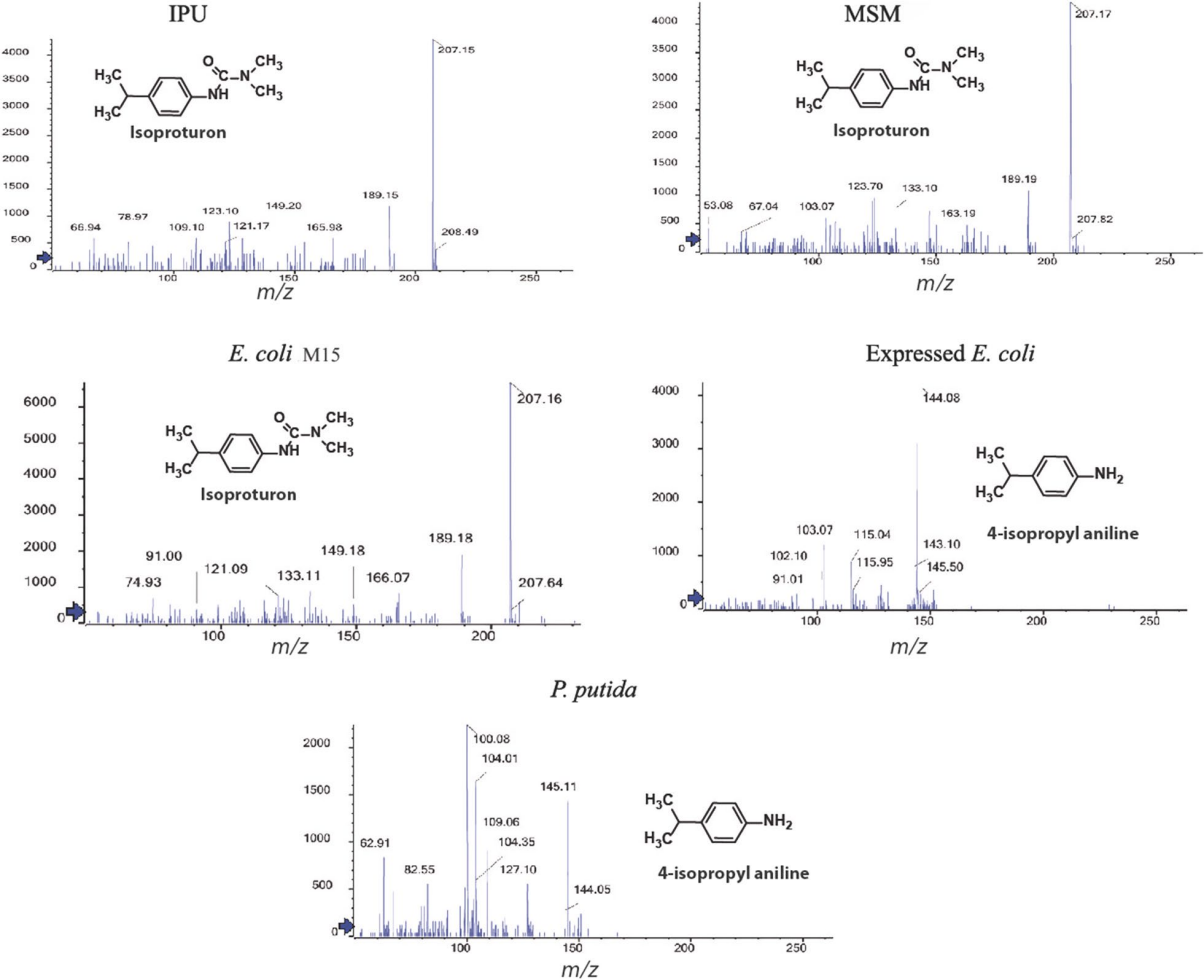
LC mass Chromatogram of IPU and its derivatives after treatment. MSM: free media containing IPU, *E. coli* M15 inoculation with *E. coli* M15, Expressed *E. coli* inoculation with expressed *E. coli*, *P. putida* inoculation with *P. putida* and IPU used as isoproturon reference standard

The biodegradation effects of IPU inoculated with different bacterial strains were tested by the identification of IPU and its derivatives using LC/MS. As a result, control (MSM medium without bacteria) and *E. coli* M15 (non- transformed) treatment provided the most abundant molecular ion ([M + H] +  = 207 m/z) that is observed at 11.7 min (Fig. 7) in comparison with IPU standard. However, different patterns were observed in samples treated with expressed *E. coli* and *P. putida* treatments, the MS spectra from [M] + of expressed *E. coli* and *P. putida* treatment generated dominant peaks and were found RT 8.3 min where the major fragment ions were *m*/*z* 144 which corresponds to 4-isopropyl-aniline at *m*/*z* 144. Surprisingly, no IPU was detected in both treatments compared to respective controls (Fig. 7).

### Gene expression analysis of *catA* using qPCR

**Fig. 8:**
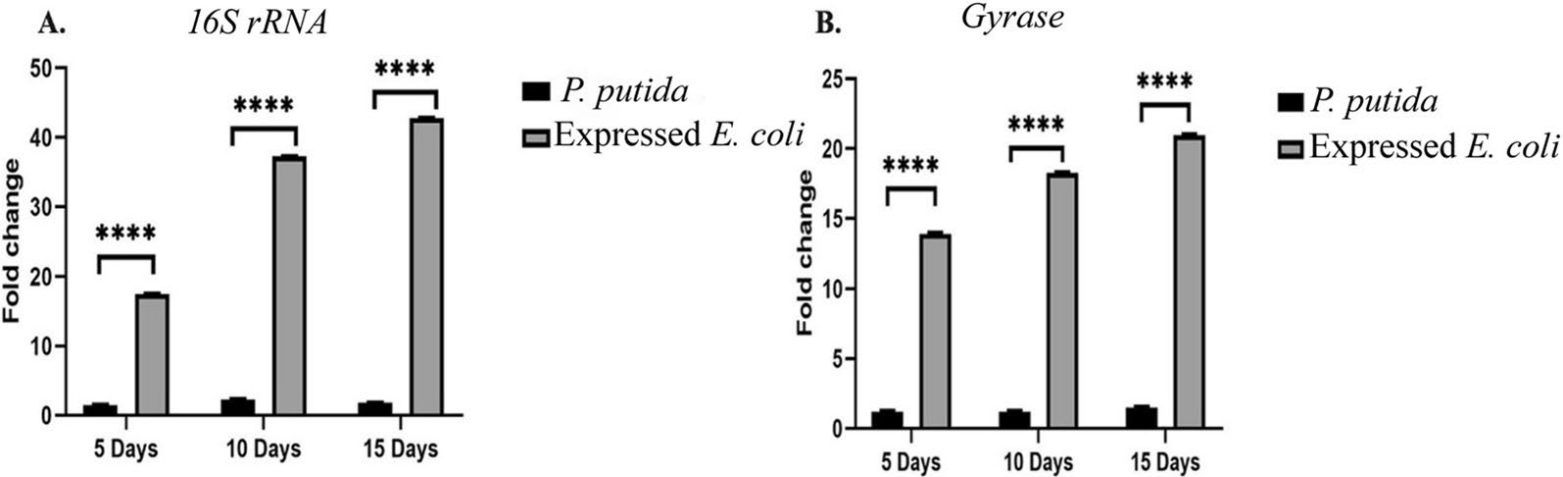
The expression levels of *catA* gene. The qRT-PCR analysis results measure the relative mRNA levels of *catA* gene of *P. putida* and expressed *E. coli*. Bars represent mean values ± standard error. **** mean significant fold change. **A** Using *16S rRNA* as housekeeping gene. **B** Using *gyrase* as housekeeping gene

The expression of *catA* gene from *P. putida* and expressed *E. coli* was measured and compared at 5,10 and 15 days of inoculation, *catA* in expressed *E. coli* showed high expression in the first 5 days of inoculation and as the incubation period increased, the gene expression increased. The relative levels of *catA* gene expression were estimated by the 2^−∆∆Ct^ method using the *16 s rRNA* and *gyrase* genes as reference gene, averaged from three independent experiments (Fig. 8).

### The effect of degradation derivatives on weeds germination

**Fig. 9:**
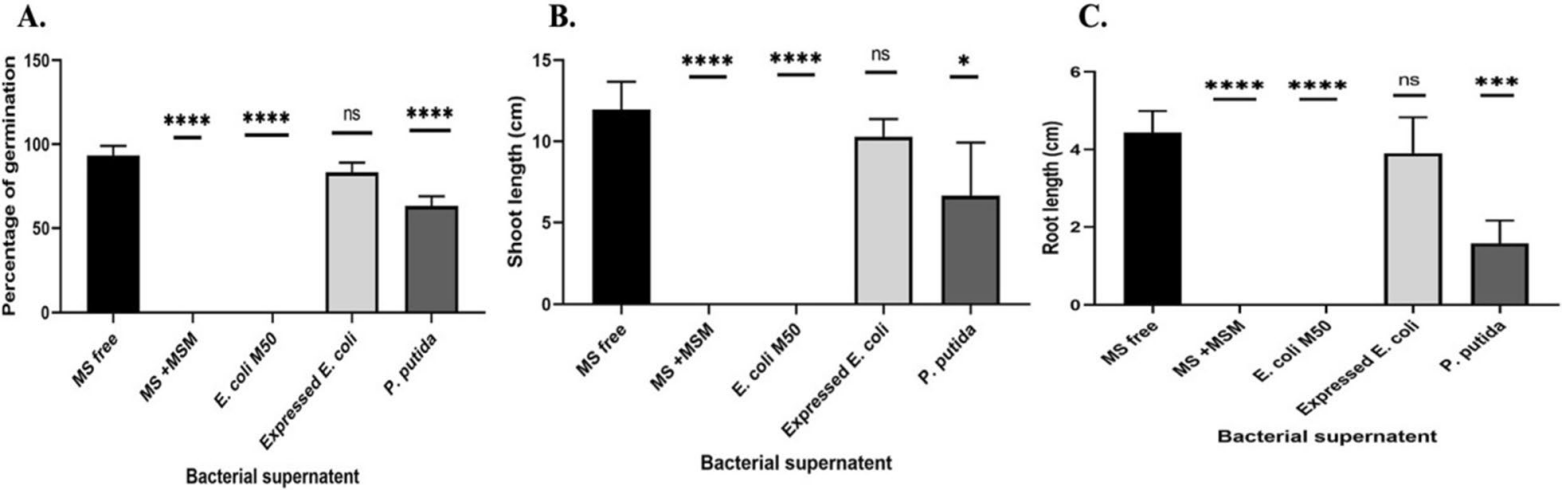
The effect of different bacterial supernatant on (**A**) weeds seeds germination, (**B**) shoot length and (**C**) root length

To assess the ability of *P. putida* and expressed *E. coli* to manipulate IPU, two different weed seeds (*Capsella bursa-pastoris* and *Phalaris canariensis*) were used. The weed seeds were sterilized and germinated on MS media supplemented with different bacterial supernatants. The data indicated that the seeds only germinated on MS free, MS with *P. putida* and MS with expressed *E. coli* (Additional file 1: Fig. S6). The result showed that the germination percentage, shoot and root length were positively affected when the *P. putida* and expressed *E. coli* were treated with IPU (Fig. 9).

## Discussion

Microorganisms have been reported as an important method responsible for the biodegradation of IPU and many other herbicides (Sun et al. 2009; Hussain et al. 2011; Huang et al. 2018). In this research, ten bacterial isolates were isolated from wheat IPU treated soil that was able to mineralize IPU. The high bacterial isolate of mineralized IPU was identified as *P. putida* according to the morphological and the molecular identification. According to a phylogenetic analysis using *16S rRNA*, the *P. putida* strain was shown to share up to 88.17% of its sequence with *P. putida* strain c58. It was also found to be clustered with other *Pseudomonas* strains. The phylogenetic tree that was constructed based on the *16S rRNA* divided into three main clusters including reference strains of *Pseudomonas*. This reflects the high similarity between the strain isolated in this study and the authenticated *Pseudomonas* strains on NCBI databases. IPU has been demonstrated to be mineralized by several *Pseudomonas* family bacterial isolates, including *P. aeruginosa* strain JS-11 (Dwivedi et al. 2011) and *Pseudomonas* sp. (Duc HD et al. 2022). Catechol dioxygenases are key intermediate enzymes that perform a fundamental role in the bacterial biodegradation of aromatic phenol herbicides; they are responsible for the aromatic ring cleavage of catechol, which is considered an important point of IPU biodegradation pathway (Sun et al. 2009; Hussain et al. 2011; Aravind et al. 2021).*P. putids catA* gene was isolated and overexpressed into *E. coli* M15. The expressed *E. coli* showed a high biodegradation efficiency compared with the *P. putida* wild-type. Biodegradation efficiency was determined using HPLC by determining the remaining IPU after the incubation period, it was observed that at first five days, the efficiency of expressed *E. coli* degradation was slow in comparison to *P. putida*, which could demonstrate the lag phase while it got accelerated as the incubation performed, most probable due to enzymes activation in the inoculated cultures (Giri et al. 2017). In this study, gene expression analysis of cloned *catA* was compared to *catA* expression from *P. putida* using qPCR. The results displayed a significant increase in gene expression of expressed *E. coli* compared with the *P. putida catA* gene expression in all studied times. Although the biodegradation efficiency of expressed *E. coli* was less than in *P. putida* for the first 5 days; the level of mRNA determined by qPCR was higher in expressed *E. coli* and this may be due to the enzyme activity and the catabolize repression. The expression of IPU mobilized genes was reported in many plants including *Arabidopsis thaliana* (Islam et al. 2021), *N. tabacum* (Wang et al. 2008), Alfalfa*, A. thaliana* and tobacco (Wang et al. 2005) and insect such as *Plutella xylostella* L. (Li et al. 2022). Santero and Díaz (2020) reported that catabolic pathways may be controlled by a regulatory circuit, resulting in a catabolize repression phenomenon that prevents their expression. Catechol 1,2dioxygenase was isolated, purified to 1.4 fold with 66.8% yield and 1.8 fold with 61.5% yield from *P. putida* and *E. coli* respectively. Purified enzyme displayed unique band in SDS PAGE with 34.26 kD corresponding to results obtained by in previous reports (Al-Hakim et al. 2015; Setlhare et al. 2018). However, the recombinant catechol 1, 2 dioxygenase enzyme isolated from *Paracoccus sp* by Aravind et al., (2021) showed a molecular size 38.6 kDa. Purified catechol 1,2 dioxygenase enzyme showed high affinity to catechol substrate in comparison to enzyme isolated from *Stenotrophomonas maltophilia* strain KB2 and purified by Guzik et al., (2013) which had Km 12.8 μM and V max 1, 218.8 U/mg of protein and enzyme isolated from *Pseudomonas 4 Chlororaphis* strain UFB2 and purified by Setlhare et al., (2018) had Km 35.76 µM and Vmax 16.67 U/mg. On the other hand, the enzyme isolated and purified from *Bacillus subtillus* showed high affinity to the substrate where Km 2.7 µg and Vmax 178U/mg protein (Abdelhaleem et al. 2019).

IPU degradation products include DDIPU, MDIPU and 4IA (Lehr et al. 1996; Sun et al. 2009). The biodegradation of the IPU was tested using the LC–MS technique. After the incubation period (15 days), the purified and concentrated IPU and its derivatives were subjected to LC–MS analysis. The results indicated that both expressed *E. coli* and *P. putida* treatments enhance the potential biodegradation of IPU. In agreement with the natural biodegradation of IPU the LC–MS data showed and extensive degradation of IPU generating 4-isopropyl-aniline compared to respective controls. These data indicated that both expressed *E. coli* and *P. putida* treatments displayed similar potential degradation of IPU pesticide in agreement with the natural degradation of IPU herbicide in biodegradation in soil (Lehr et al. 1996; Sorensen et al. 2003; Sun et al. 2009). However, several soil isolated strains have shown a high ability to degrade the *N*, *N*-dimethyl phenylurea herbicides including IPU (Tixier et al. 2000). In this context, different sources are identified as environmental aniline-based compounds such as plastics, dyes, and pharmaceutical-derived products. In general, aniline-based compounds are relatively easily degraded and completely mineralized by microorganisms as it has been previously described by Parris (1980). IPU biodegradation to 4-isopropylaniline in the soil may significantly minimize the mineralization of the herbicide molecule due to the absorption of the aniline metabolite into the soil (Johannesen et al. 2003). In conclusion, to improve the bioremediation and biodegradation process in the environment, it’s important to study and understand the herbicides biodegradation. The results of this research may reveal that bacterial isolate isolated from IPU treated soil *P. putida* possesses an effective catalytic enzyme system for IPU degradation. In addition, the expressed *E. coli* with *catA* gene showed a significant increase in IPU degradation. So such bacterial isolates could be valuable for bioremediation and biodegradation of herbicides in polluted water and soil environments.

### Supplementary Information

**Additional file 1: Table S1.** The primers sequences, expected size, annealing temperature and references utilized in this study. **Table S2.** The morphological characters of IPU-FACU1 and IPU-FACU7 bacterial isolates. **Fig S1.** The expression vector pQ30-*catA *with *catA *ORF (936 bp). **Fig S2.** The CFU values of different bacterial isolates (IPU-FACU1 to IPU-FACU10) in MSM media including 200 mg/L of IPU. The data are shown as the mean standard error of the three replicate samples. **Fig S3.** The PCR amplification of *16 sRNA* gene, M is 1 kb DNA ladder markers, line1: FACU1 isolate and line 2: FACU7 isolate. **Fig S4.** The PCR amplification of *catA* gene, M is 1 kb DNA ladder markers, line1: *P. putida* isolate and line 2: *A. johnsonii*. **Fig S5.** The SDS-PAGE for the catA protein induction from *E. coli *expressed* P.putida catA *gene. A: using different IPTG concentrations (Lane M is PiNK prestained protein leader; lane 1 is the expressed *E. coli* after induction with 1 M IPTG after 3 h; lane 2, 3 and 5 is non- induction expressed *E. coli* after 3 h; lane 4 is the expressed *E. coli* after induction with 0.5 M IPTG after 3 h; lane 6 is the expressed *E. coli* after induction with 0.1 M IPTG after 3 h and lane 7 is non- induction expressed *E. coli* after zero time); B: using different incubation times (Lane M is PiNK prestained protein leader; lane 1 is the non-induction expressed *E. coli* at 37 °C; lane 2 is the expressed *E. coli* after 30 minutes induction; lane 3 is the expressed *E. coli* after 1 h induction; lane 4 is the expressed *E. coli* after 1.5 h induction; lane 5 is the expressed *E. coli* after 2 h induction; lane 6 is the expressed *E. coli* after 3 h induction and lane 7 is the expressed E. coli after 4 h induction) and C: using different temperatures (Lane M is PiNK prestained protein leader; lane 1 is the non-induction expressed *E. coli* at 37 °C; lane 2 is the expressed E. coli at 20 °C; lane 3 is the expressed *E. coli* at 28 °C; lane 4 is the expressed E. coli at 30 °C; lane 5 is the expressed E. coli at 37 °C; lane 6 is the expressed *E. coli* at 40 °C and lane 7 is the expressed *E. coli* at 45°C). The protein samples were run on 15%SDS– PAGE gel and visualized by staining with Coomassie blue. **Fig S6.** The effect of IPU degradation by bacterial strains on weeds germination. A: *Phalaris canariensis seeds *and* B: Capsella bursa-pastoris.*

## Data Availability

Not applicable.

## Abbreviations

*E.** coli*: *Escherichia coli*
*P.** putida*: *Pseudomonas putida*
IPU: Isoproturon: catechol 1,2-dioxygenase gene: catA gene
HPLC: High-performance liquid chromatography
qPCR: Quantitative polymerase chain reaction
HILIC: Hydrophilic interaction chromatography
MDIPU: 1-(4-Isopropylphenyl)-3-methylurea
4-IA: 4-Isopropylaniline
DDIPU: 1-(4-Isopropylphenyl) urea
MIC: Minimum inhibitory concentration
CFU: Colony forming unit
LB: Luria Broth
PCR: Polymerase chain reaction
NCBI: National center for biotechnology information
OPF: Open reading frame
SDS-PAGE: Sodium dodecyl sulfate–polyacrylamide gel electrophoresis
OD: Optical density
Km: Michaelis–Menten constant
V max: Maximum velocity
CCMA: Cis, cis-muconic acid
SPE: Solid-Phase Extraction
LC–ESI–MS/MS: Liquid chromatography-electrospray ionization-tandem mass spectrometry
ANOVA: Electrospray ionization: ESI, a one-way analysis of variance
DDBJ: DNA Data Bank of Japan
IPTG: Isopropyl β-D-1-thiogalactopyranoside
*A.** thaliana*: *Arabidopsis thaliana*
