# Supplementary material for: Biodegradation of isoproturon by Escherichia coli expressing a Pseudomonas putida catechol 1,2-dioxygenase gene
Source: AMB Express. 2023 Sep 26;13:101. doi: 10.1186/s13568-023-01609-9 (PMC10522561; doi:10.1186/s13568-023-01609-9)
Supplement: Supplementary file 1 — Additional file 1: Table S1. The primers sequences, expected size, annealing temperature and references utilized in this study. Table S2. The morphological characters of IPU-FACU1 and IPU-FACU7 bacterial isolates. Fig S1. The expression vector pQ30-catA with catA ORF (936 bp). Fig S2. The CFU values of different bacterial isolates (IPU-FACU1 to IPU-FACU10) in MSM media including 200 mg/L of IPU. The data are shown as the mean standard error of the three replicate samples. Fig S3. The PCR amplification of 16 sRNA gene, M is 1 kb DNA ladder markers, line1: FACU1 isolate and line 2: FACU7 isolate. Fig S4. The PCR amplification of catA gene, M is 1 kb DNA ladder markers, line1: P. putida isolate and line 2: A. johnsonii. Fig S5. The SDS-PAGE for the catA protein induction from E. coli expressed P.putida catA gene. A: using different IPTG concentrations (Lane M is PiNK prestained protein leader; lane 1 is the expressed E. coli after induction with 1 M IPTG after 3 h; lane 2, 3 and 5 is non- induction expressed E. coli after 3 h; lane 4 is the expressed E. coli after induction with 0.5 M IPTG after 3 h; lane 6 is the expressed E. coli after induction with 0.1 M IPTG after 3 h and lane 7 is non- induction expressed E. coli after zero time); B: using different incubation times (Lane M is PiNK prestained protein leader; lane 1 is the non-induction expressed E. coli at 37 °C; lane 2 is the expressed E. coli after 30 minutes induction; lane 3 is the expressed E. coli after 1 h induction; lane 4 is the expressed E. coli after 1.5 h induction; lane 5 is the expressed E. coli after 2 h induction; lane 6 is the expressed E. coli after 3 h induction and lane 7 is the expressed E. coli after 4 h induction) and C: using different temperatures (Lane M is PiNK prestained protein leader; lane 1 is the non-induction expressed E. coli at 37 °C; lane 2 is the expressed E. coli at 20 °C; lane 3 is the expressed E. coli at 28 °C; lane 4 is the expressed E. coli at 30 °C; lane 5 is the expres [file 13568_2023_1609_MOESM1_ESM.docx]

**Table S1: The primers sequences, expected size, annealing temperature and references utilized in this study**

| **Purpose** | **Gene** | **Sequence (5’-3’)** | **Expected size** | **Annealing Temp. (°C)** | **Reference** |
| --- | --- | --- | --- | --- | --- |
| Bacterial identification | *16S rRNA* | 27F: AGAGTTTGATCMTGGCTCAG | 1520 | 56 | Frank et al. 2008; Abdelhadi et al. 2016 |
|  |  | 1492R: TACGGYTACCTTGTTACGACTT |  |  |  |
| *catA* gene amplification | *catA* | catA-F: ACGATGACCGTGAAAATTTCC | 936 | 56 | This study |
|  |  | catA-R: CCTGCCAGTAGCGTTGCA |  |  |  |
| *catA* gene cloning and expression | *catA* | catA-BamHI: TCAGGATCCATGACCGTGAAAATTTCCCACACTG | 1200 | 58 | This study |
|  |  | catA-HindIII: TTGAAGCTTTCAGTGGTGGTGGTGGTGGTGGTGGCCCTCCTGCAAACGCCCGCGG |  |  |  |
| qPCR | *catA* | F: GTGATGTTCCTTCAGGGCCA | 158 | 56 | This study |
|  |  | R: TCGGCATCGGTGATGATACG |  |  |  |
| qPCR | *16S rRNA*  (reference gene) | F: ACTCCTACGGGAGGCAGCAGT | 198 | 56 | Clifford et al. 2012 |
|  |  | R: TATTACCGCGGCTGCTGGC |  |  |  |
| qPCR | *Gyrase B*  (reference gene) | F: CGATTGTGTCCGTTAAAGTGC | 148 | 56 | Wang et al. 2016 |
|  |  | R: TGCAAACGCTCACCAACTG |  |  |  |

**Table S2: The morphological characters of IPU-FACU1 and IPU-FACU7 bacterial isolates**

| **Bacterial isolates** | **IPU-FACU1** | **IPU-FACU7** |
| --- | --- | --- |
| Gram stain | Negative | Negative |
| Colony color | Cream | Cream |
| Shape | Rod | Rod |
| Capsule | - | - |
| Spore | - | - |
| Motility | + | - |
| Catalase | + | + |
| Citrate | + | - |
| Oxidase | + | - |
| Indole producttion | - | - |
| Urease dihydrolase | - | - |
| Anaerobic growth | + | + |
| Voges-Proskauer (VP) | - | - |
| Methyl red (MR) | - | - |
| Blood hemolysis | - | - |
| Hydrolyses of starch | - | - |
| Growth at 4 °C | - | - |
| Growth at 30 °C | + | + |
| Growth at 37 °C | + | + |
| Growth at 40 °C | + | + |
| Growth at 44 °C | - | - |


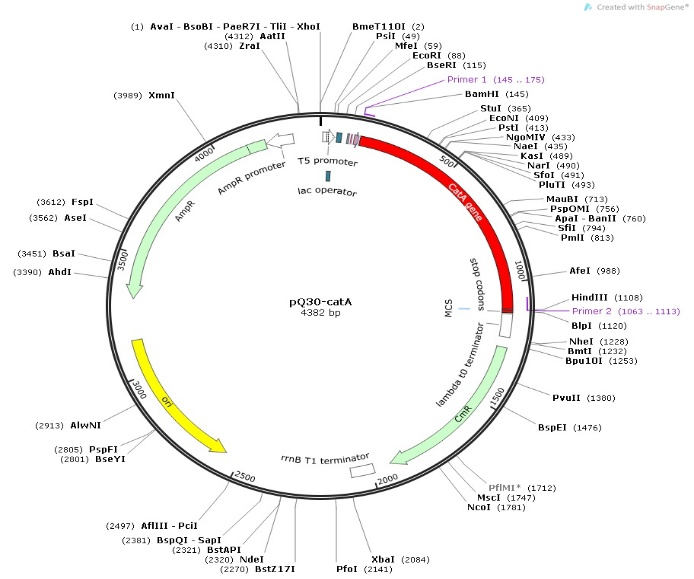


**Fig. S1: The expression vector pQ30-*catA* with *catA* ORF (936 bp).**


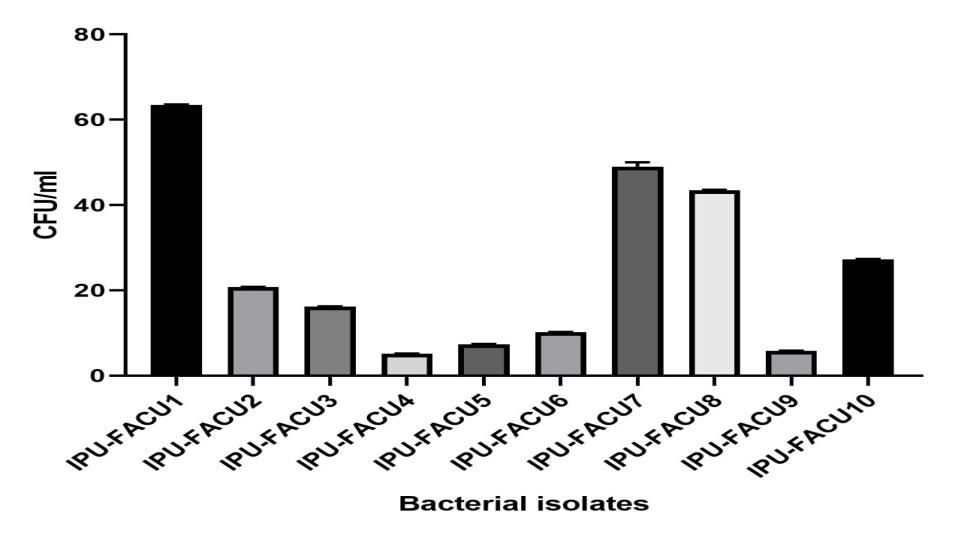


**Fig. S2: The CFU values of different bacterial isolates (IPU-FACU1 to IPU-FACU10) in MSM media including 200 mg/L of IPU. The data are shown as the mean standard error of the three replicate samples.**


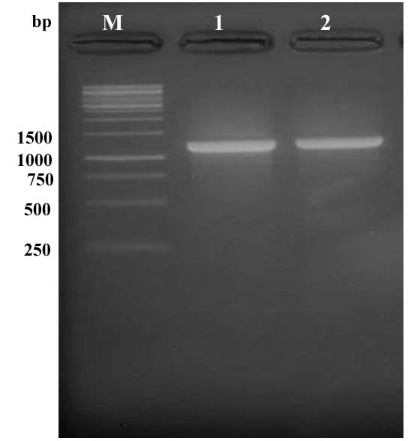


**Fig. S3: The PCR amplification of *16 sRNA* gene, M is 1 kb DNA ladder markers, line1: FACU1 isolate and line 2: FACU7 isolate**


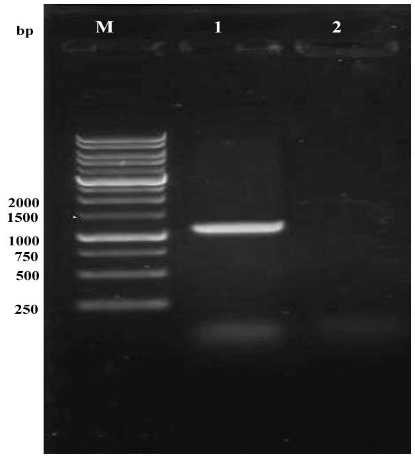


**Fig. S4: The PCR amplification of *catA* gene, M is 1 kb DNA ladder markers, line1: *P. putida* isolate and line 2: *A. johnsonii***


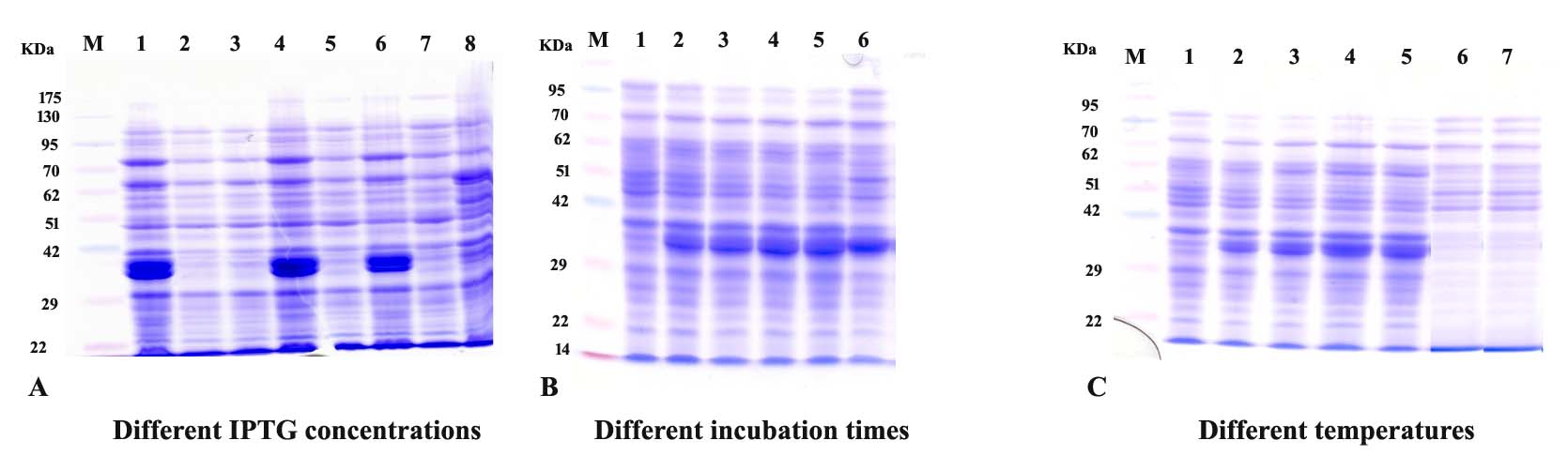


**Fig. S5: The SDS-PAGE for the catA protein induction from *E. coli* expressed *P.putida catA* gene. A: using different IPTG concentrations (Lane M is PiNK prestained protein leader; lane 1 is the expressed *E. coli* after induction with 1 M IPTG after 3 h; lane 2, 3 and 5 is non- induction expressed *E. coli* after 3 h; lane 4 is the expressed *E. coli* after induction with 0.5 M IPTG after 3 h; lane 6 is the expressed *E. coli* after induction with 0.1 M IPTG after 3 h and lane 7 is non- induction expressed *E. coli* after zero time); B: using different incubation times (Lane M is PiNK prestained protein leader; lane 1 is the non-induction expressed *E. coli* at 37 °C; lane 2 is the expressed *E. coli* after 30 minutes induction; lane 3 is the expressed *E. coli* after 1 h induction; lane 4 is the expressed *E. coli* after 1.5 h induction; lane 5 is the expressed *E. coli* after 2 h induction; lane 6 is the expressed *E. coli* after 3 h induction and lane 7 is the expressed E. coli after 4 h induction) and C: using different temperatures (Lane M is PiNK prestained protein leader; lane 1 is the non-induction expressed *E. coli* at 37 °C; lane 2 is the expressed E. coli at 20 °C; lane 3 is the expressed *E. coli* at 28 °C; lane 4 is the expressed E. coli at 30 °C; lane 5 is the expressed E. coli at 37 °C; lane 6 is the expressed *E. coli* at 40 °C and lane 7 is the expressed *E. coli* at 45°C). The protein samples were run on 15%SDS– PAGE gel and visualized by staining with Coomassie blue.**


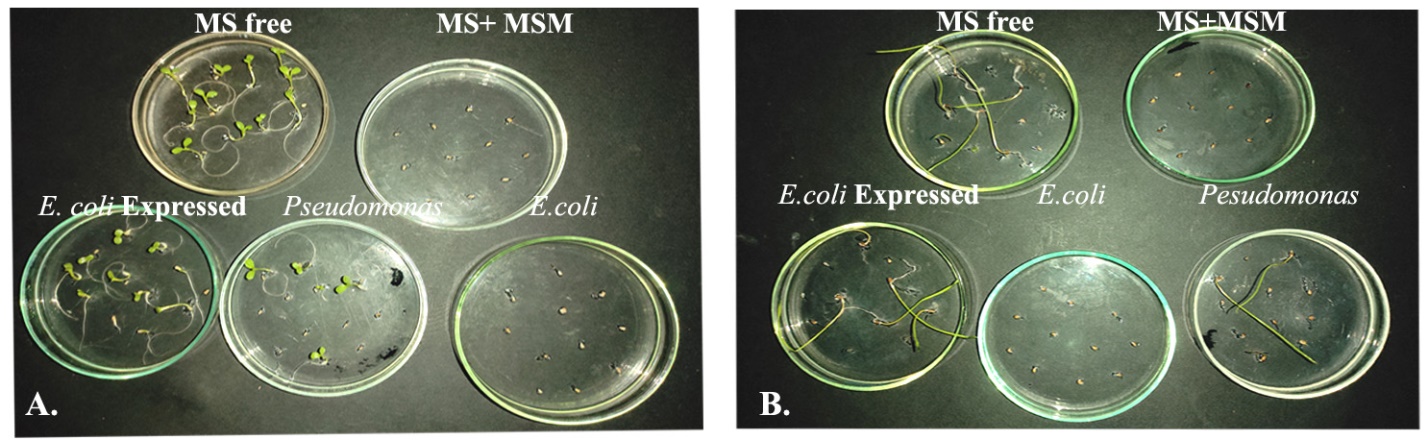


**Fig. S6: The effect of IPU degradation by bacterial strains on weeds germination. A: *Phalaris canariensis seeds* and *B: Capsella bursa-pastoris.***
